# Case Report: Targeting EGFR exon 20 insertion and MTAP loss in refractory metastatic upper tract urothelial carcinoma

**DOI:** 10.3389/fonc.2026.1928596

**Published:** 2026-08-27

**Authors:** Karen Hoi Lam Li, Roland Ching Yu Leung, Thomas Yau, Joshua Jing Xi Li, Josephine Wing Yan Tsang, Jenny Wai Yan Lo, Gerry Gin Wai Kwok, Jeffrey Sum Lung Wong, Alvin Hoi Wai Chan, Bryan Cho Wing Li

**Affiliations:** 1 Department of Medicine, Queen Mary Hospital, The University of Hong Kong, Hong Kong, Hong Kong SAR, China; 2 Centre of Cancer Medicine, The University of Hong Kong, Hong Kong, Hong Kong SAR, China; 3 Department of Pathology, Queen Mary Hospital, The University of Hong Kong, Hong Kong, Hong Kong SAR, China

**Keywords:** amivantamab, EGFR exon 20 insertion, MTAP loss, precision oncology, upper tract urothelial carcinoma

## Abstract

This case report illustrates the use of amivantamab in combination with pemetrexed-based chemotherapy for the treatment of a 60-year-old man with refractory metastatic upper tract urothelial carcinoma (UTUC) with epidermal growth factor receptor (EGFR) exon 20 insertion (D770_N771insGF) and methylthioadenosine phosphorylase (MTAP) loss identified in both the primary left nephroureterectomy tissue and lung metastasis biopsy. The patient, who had progressed on five prior lines of therapy including platinum-based chemotherapy, immunotherapy, and antibody-drug conjugates, attained a sustained partial response with favourable tolerability before eventual disease progression with brain metastasis. This case is unique because of the uncommon molecular profile, with an EGFR exon 20 insertion observed in only 0.7% of urothelial carcinomas, which highlights the potential for repurposing targeted therapies developed for other malignancies in biomarker-defined subgroups of UTUC. This case report highlights the significance of next-generation sequencing in uncovering rare therapeutic targets, the potential of synthetic lethality approaches in MTAP-deficient tumours, and supports further research to explore molecular-based cancer classification and treatment strategies in precision oncology.

## Introduction

Upper tract urothelial carcinoma (UTUC) originates from the ureter, renal pelvis, and calyces and comprises approximately 5%–10% of all urothelial carcinomas. UTUC exhibits an aggressive biological nature, with around two-thirds of cases being invasive at diagnosis (1). Due to the relative rarity of UTUC, most treatment decisions regarding UTUC are extrapolated from urothelial bladder cancer (UBC). In addition, urothelial carcinoma with squamous cell differentiation represents a unique subtype that is often underrepresented in clinical trials (2). The identification of predictive biomarkers in patients with urothelial carcinoma has been limited by the heterogeneity of the disease and the variability of methodologies employed in biomarker research (3). The loss of methylthioadenosine phosphorylase (MTAP) and cyclin-dependent kinase inhibitor 2A (CDKN2A) in urothelial carcinoma marks a “cold” immune microenvironment and simultaneously creates exploitable vulnerability to antifolates (4, 5). EGFR exon 20 insertions are reported in only 0.7% of urothelial carcinomas and may represent a rare subgroup of patients who may benefit from EGFR-targeted therapies (6). Amivantamab monotherapy or in combination with chemotherapy has shown impressive results in non-small-cell lung cancer with EGFR exon 20 insertions (7, 8). We present the case of a 60-year-old man with heavily pretreated, refractory UTUC with multiple lung metastases harbouring MTAP loss, CDKN2A/cyclin-dependent kinase inhibitor 2B (CDKN2B) loss, and an EGFR exon 20 insertion who received amivantamab plus pemetrexed-based chemotherapy as the sixth line of therapy, with a treatment duration of 10 months. To our knowledge, this is the first case report of amivantamab being used to target urothelial carcinoma with an EGFR exon 20 insertion.

## Case description

**Figure 1:**
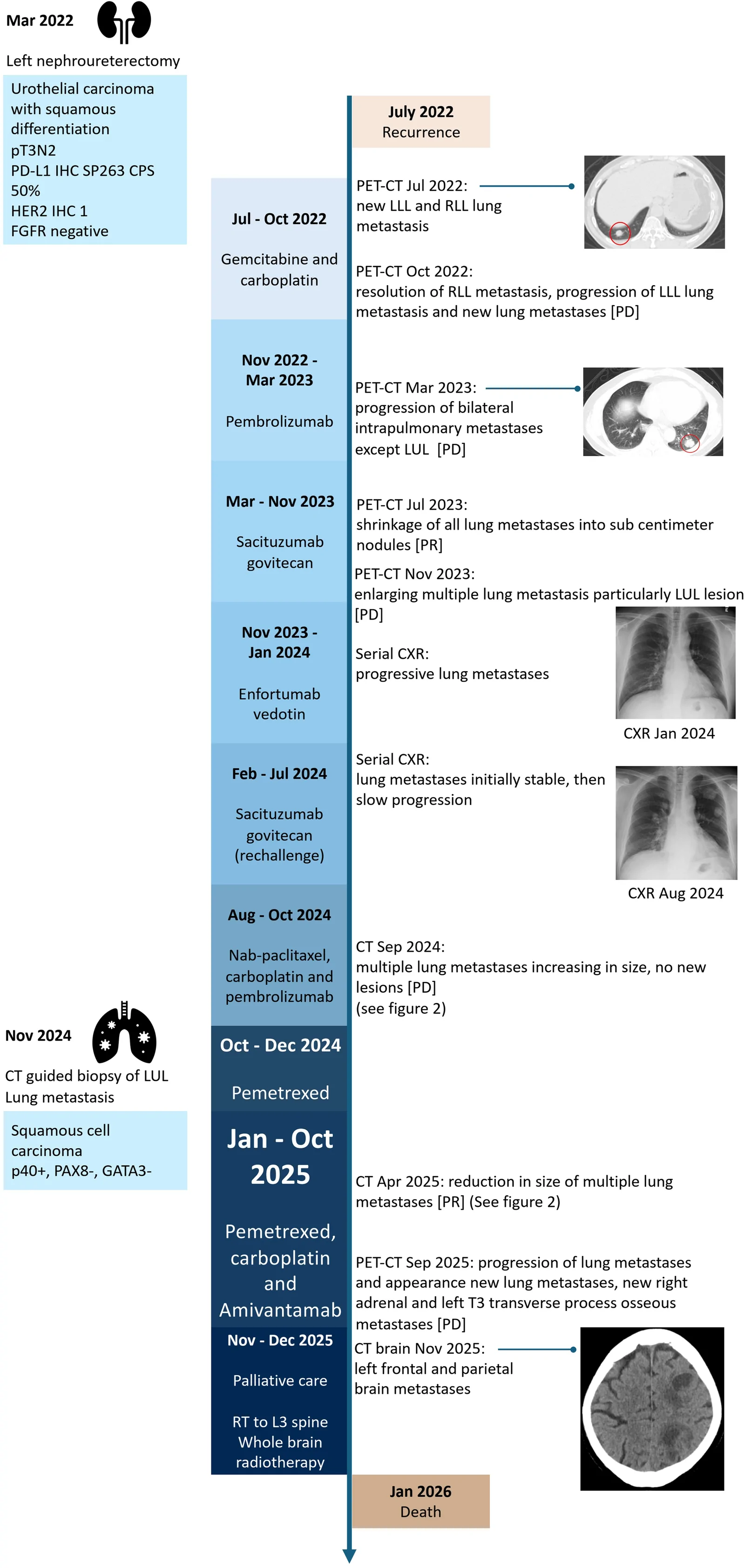
Treatment timeline. The patient was assessed using PET-CT or CT imaging according to RECIST v 1.1 criteria. PET-CT, positron emission tomography-computed tomography; CT, computed tomography; CXR, chest X-ray; PD, progressive disease; SD, stable disease; LLL, left lower lobe; LUL, left upper lobe; RLL, right lower lobe.

**Figure 2:**
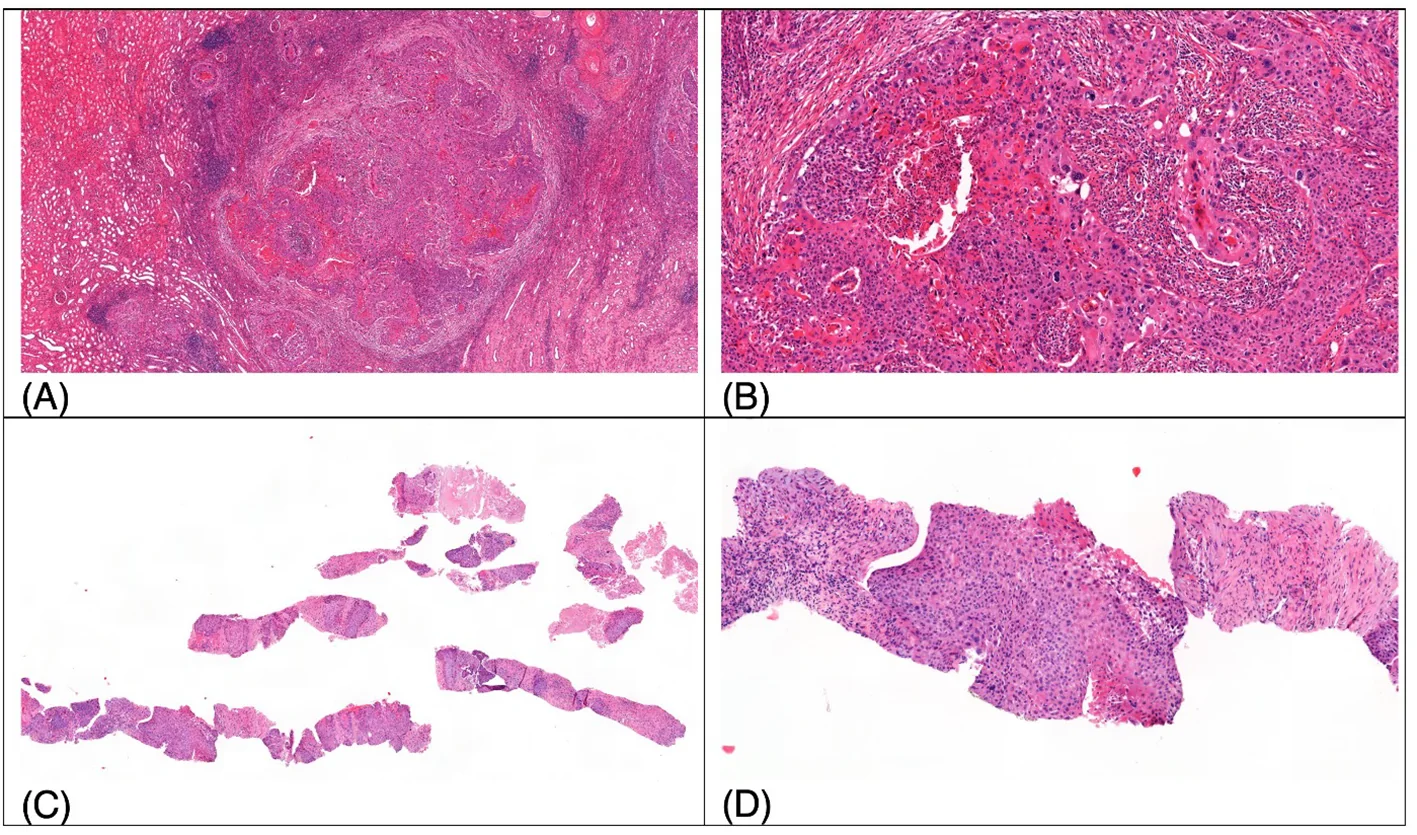
Representative histopathology sections. **(A)** Sheets of tumour cells invading the renal parenchyma (magnification: × 5). **(B)** Tumour cells showing marked nuclear enlargement, pleomorphism, and hyperchromasia (magnification: × 20). **(C)** Cores of lung tissue infiltrated by tumour cells (magnification: × 5). **(D)** Tumour cells displaying similar morphology to that of the nephrectomy specimen (magnification: × 20).

A 60-year-old Asian man presented with lower urinary tract symptoms and elevated prostate-specific antigen (PSA). Contrast magnetic resonance imaging (MRI) of the pelvis in June 2019 showed two PI-RADS 3 lesions. Prostate biopsy performed in October 2019 showed no malignancy. Prostate-specific membrane antigen (PSMA) positron emission tomography–computed tomography (PSMA PET-CT) for increasing PSA in March 2021 incidentally found a hypermetabolic left renal mass and lymphadenopathies along the left para-aortic chain at L2, L2/3, and L3, without evidence of distant metastasis. Computed tomography urography performed in March 2021 showed a left renal lower pole infiltrative mass and left para-aortic nodes. He underwent left nephroureterectomy in March 2022 (**Figure 1**). Postoperative pathological assessment confirmed urothelial carcinoma with squamous differentiation with multiple regional nodal metastases (pT3N2), and the PD-L1 immunohistochemistry (IHC) SP263 CPS score was 50% (**Figures 2A, B**). Adjuvant chemotherapy was offered per multidisciplinary team (MDT) discussion, but the patient postponed his medical oncology clinic appointment because he had to travel abroad for work. Restaging PET-CT in July 2022 showed two new hypermetabolic nodules in the bilateral posterior lung bases, measuring 1.1 cm on the left and 1.5 cm on the right, suggestive of intrapulmonary metastases. During the MDT discussion, systemic therapy was proposed instead of surgical resection for intrapulmonary metastases in view of the short disease-free interval. Further molecular testing with fibroblast growth factor receptor 3 (FGFR3) genetic alterations and Human epidermal growth factor receptor 2 (HER2) IHC testing were performed in accordance with regulatory approvals for erdafitinib and fam-trastuzumab deruxtecan. Molecular testing of the left nephroureterectomy tissue showed a HER2 IHC score of 1 and the absence of FGFR3 alteration. Comprehensive molecular profiling was not pursued after the MDT discussion at that time because therapies for other genomic alterations were mostly investigational. He had an Eastern Cooperative Oncology Group (ECOG) performance status of 1 and an estimated glomerular filtration rate (eGFR) of 30–40 mL/min postoperatively. The patient was ineligible for cisplatin due to impaired renal function.

He received gemcitabine and carboplatin as the first-line treatment for metastatic urothelial carcinoma in July 2022, which was the standard of care at the time (9). Reassessment PET-CT three months later in October 2022 showed resolution of the right lung base pulmonary metastasis but progression of the left lung base metastasis and emergence of multiple new intrapulmonary metastases. He was then switched to pembrolizumab in November 2022 (10). Interval PET-CT 4 months later in March 2023 confirmed progression of the bilateral intrapulmonary metastases. Upon disease progression after platinum-based chemotherapy and immunotherapy, sacituzumab govitecan was offered after an MDT meeting based on phase II data (11). Reassessment PET-CT 4 months after treatment with sacituzumab govitecan showed regression of preexisting bilateral intrapulmonary metastases, with only residual subcentimetre nodules, corresponding to a partial response according to Evaluation Criteria in Solid Tumours version 1.1 (RECIST v1.1), although repeat PET-CT after 8 months of treatment in November 2023 showed evidence of growth of multiple lung metastases. He received enfortumab vedotin for 2 months until chest X-ray (CXR) showed frank enlargement of lung metastases in February 2024 (12). Further treatment options including taxanes and palliative care were discussed. The patient was keen to pursue further anticancer therapy, and subsequently a decision was made to rechallenge with sacituzumab govitecan given the previous dramatic response after MDT discussion. Serial on-treatment CXRs initially showed stable lung metastases; however, enlarged pulmonary metastases were observed in August 2024, leading to cessation of sacituzumab govitecan after 6 months. Conventional treatment options were exhausted, and he had no susceptible FGFR3 genetic alterations nor HER2 amplification. The patient and family were counselled on the treatment options again, and after extensive discussion, they opted for taxane-based chemotherapy. They were also keen to undergo a lung metastasis biopsy to identify rare druggable mutations. In light of the encouraging result of the chemoimmunotherapy combination for refractory metastatic urothelial carcinoma in the phase II PEANUT study, a combination of carboplatin, nab-paclitaxel, and pembrolizumab was recommended in the MDT meeting and discussed with the patient and his family (13). The expected limited efficacy of this combination regimen and potential toxicities were accepted by the patient. Reassessment computed tomography (CT) with contrast in September 2024 showed multiple lung metastases up to 4.1 cm in the left upper lobe, 3.1 cm in the right upper lobe, 5.6 cm in the right lower lobe, and 3.7 cm in the left lower lobe; all were enlarged compared with the previous PET-CT in November 2023. As the patient continued to have an ECOG performance status of 1 and was keen to receive active treatment if available, a CT-guided left upper lobe lung biopsy was performed in November 2024, and next-generation sequencing (NGS) was performed on the initial left nephroureterectomy specimen in August 2024 and the lung biopsy using FoundationOne^®^CDx in November 2024. The lung biopsy result was consistent with squamous cell carcinoma; immunohistochemical staining was positive for p40 and negative for PAX8 and GATA3 (**Figures 2C, D**). NGS of the left nephroureterectomy specimen revealed an EGFR exon 20 insertion (D770_N771insGF) and amplification, MTAP loss, CDKN2A loss, CDKN2B loss, phosphatidylinositol-4,5-bisphosphate 3-kinase catalytic subunit alpha (PIK3CA) T1052K, R93Q, KLHL6 A9T, and TP53 loss exons 10–11. NGS of lung metastasis after disease progression demonstrated identical genomic findings to those in the left nephroureterectomy tissue, supporting metastasis from upper tract urothelial carcinoma (**Table 1**).

**Table 1:** Next-generation sequencing of left nephroureterectomy tissue and left upper lobe lung metastasis biopsy using FoundationOne^®^CDx.

Sample	Left nephroureterectomy tissue (March 2022)	Left upper lobe lung metastasis biopsy (November 2024)
Genomic findings	**EGFR** exon 20 insertion (D770_N771insGF), amplification; **MTAP** loss; **CDKN2A** loss; **CDKN2B** loss; **PIK3CA** T1052K, R93Q; **KLHL6** A9T; **TP53** loss exons 10–11	**EGFR** exon 20 insertion (D770_N771insGF), amplification; **MTAP** loss; **CDKN2A** loss; **CDKN2B** loss; **PIK3CA** T1052K, R93Q; **KLHL6** A9T; **TP53** loss exons 10–11
Biomarker findings	HRD signature: HRDsig negative	HRD signature: HRDsig negative
	Microsatellite status: MS-stable	Microsatellite status: MS-stable
	Tumour mutational burden: 6 Muts/Mb	Tumour mutational burden: 5 Muts/Mb

EGFR, epidermal growth factor receptor; MTAP, methylthioadenosine phosphorylase; CDKN2A, cyclin-dependent kinase inhibitor 2A; CDKN2B, cyclin-dependent kinase inhibitor 2B; PIK3CA, phosphatidylinositol-4,5-bisphosphate 3-kinase catalytic subunit alpha

**Figure 3:**
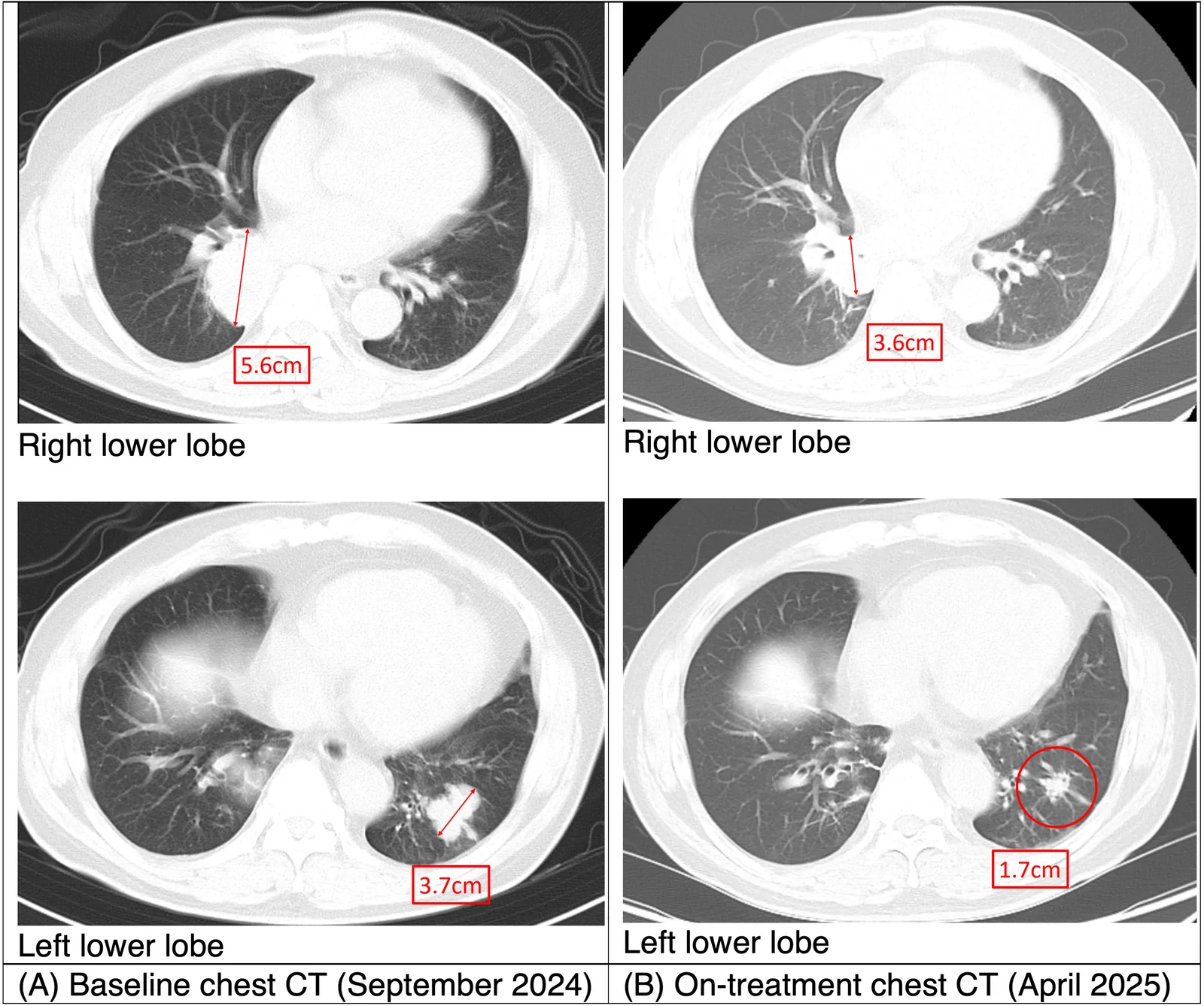
Radiological evaluation of lung metastasis by chest CT. **(A)** Pretreatment chest CT illustrates a 5.6-cm right lower lobe lung metastasis and a 3.6-cm left lower lobe lung metastasis. **(B)** On-treatment chest CT obtained 3 months after amivantamab, pemetrexed, and carboplatin shows a 3.6-cm right lower lobe lung metastasis and a 1.7-cm left lower lobe lung metastasis. CT, computed tomography.

The genomic findings were presented to the MDT tumour board for further discussion. A clinical trial for MTAP-deleted urothelial carcinoma was unavailable. The experimental use of a novel combination of pemetrexed, carboplatin, and amivantamab was recommended based on the EGFR exon 20 insertion, MTAP loss, and CDKN2A/B loss identified in both the left nephrouretectomy tissue and the lung metastasis biopsy, and the absence of alternative biomarker-directed therapy options. The rationale, possible side effects, and experimental nature of the treatment regimen were thoroughly discussed with the patient. Written informed consent was obtained from the patient regarding off-label treatment. The patient then received pemetrexed monotherapy from October to December 2024 with good tolerance and was treated with pemetrexed, amivantamab, and carboplatin from January 2025, based on the EGFR exon 20 insertion, MTAP loss, and CDKN2A/B loss identified in both the left nephrouretectomy tissue and the lung metastasis biopsy. Amivantamab 1,400 mg weekly was administered for the first 4 weeks, with 350 mg administered on cycle 1 day 1 and the remainder on cycle 1 day 2, based on his body weight of 75 kg. Amivantamab was administered at a dose of 1,750 mg every 3 weeks, starting in cycle 3. Carboplatin-pemetrexed was administered every 3 weeks, with a 20% reduction upfront and prophylactic granulocyte-colony stimulating factor (G-CSF) support. Treatment was well tolerated, with grade 1 acneiform rash (Common Terminology Criteria for Adverse Events version 5.0) treated with clindamycin solution and oral doxycycline. Carboplatin was dropped after cycle 7. Reassessment CT after 3 months of treatment showed a decrease in the size of all lung metastases without non-target lesions and new lesions, demonstrating a partial response (− 41% change from baseline) by RECIST v1.1 (**Table 2**; **Figure 3**); the largest lung metastasis in the right lower lobe shrank from 5.6 to 3.6 cm. PET-CT in September 2025 confirmed progression of the left apical, left upper lobe, and left posterior lung base nodules into sizeable intrapulmonary masses; the right lower lobe nodule enlarged to 6.5 cm, and new right adrenal and left T3 transverse process osseous metastases appeared, suggestive of overall disease progression. He received palliative radiotherapy for T3 bone metastasis in October 2025. In view of the regimen’s good tolerability and the lack of an alternative efficacious therapy, this regimen was continued for 10 months until October 2025. He was admitted to the hospital for seizures and right hemiplegia in November 2025, and computed tomography of the brain revealed left frontal and parietal brain metastases with vasogenic oedema. The patient and family opted for palliative care, and treatment was discontinued. He received whole-brain radiotherapy with dexamethasone cover. He succumbed in hospice care in January 2026.

**Table 2:** RECIST v1.1 measurements at baseline and on-treatment.

Target lesions	Baseline chest CT (September 2024)	On-treatment chest CT (April 2025)
	Pretreatment diameter (mm)	Posttreatment diameter (mm)
Lung right lower lobe	56	36
Lung right upper lobe	31	5
Lung left lower lobe	37	17
Lung left upper lobe	41	39

## Discussion

We describe a case of targeting MTAP-deleted refractory upper tract urothelial carcinoma via synthetic lethality using pemetrexed-based chemotherapy and amivantamab for an EGFR exon 20 insertion. The patient achieved a notable and durable partial response according to RECIST v1.1, with a total treatment duration of 10 months and good tolerability. To the best of our knowledge, this is the first reported case of urothelial carcinoma harbouring rare EGFR exon 20 insertions (EGFR ex20ins) treated with amivantamab.

The MTAP gene is located next to the tumour suppressor gene CDKN2A at 9p21 and is often homozygously codeleted with CDKN2A and CDKN2B in MTAP-loss tumours (14). Of all human cancers, 15% exhibit a loss of the MTAP gene (15). MTAP is deleted in 25% of urothelial carcinomas (16). MTAP-deficient urothelial carcinomas are associated with more aggressive disease and a higher risk of visceral metastasis (17). MTAP loss affects the methionine cycle by impairing the methionine and adenosine salvage pathways. Adenosine nucleotides are produced either through *de novo* biosynthesis via folate-mediated one-carbon metabolism or via the salvage pathway controlled by MTAP. Loss of MTAP confers susceptibility to synthetic lethality when treated with antifolates that inhibit *de novo* purine biosynthesis (5). A phase II clinical trial showed that pemetrexed had an overall response rate of 43% in patients with previously treated metastatic urothelial carcinoma with MTAP deficiency (three out of seven patients) (5). Preclinical models suggest that MTAP loss creates an immunosuppressive tumour microenvironment by impairing T-cell function, conferring resistance to immunotherapy (4).

In addition to FGFR3 alterations and HER2 overexpression, which are recognised as actionable biomarkers for advanced urothelial carcinoma, ongoing research has focused on identifying novel biomarkers (3). EGFR alterations are found in 4% of urothelial carcinomas, whereas EGFR exon 20 insertions are observed in only 0.7% of urothelial carcinomas (6). Common comutations with EGFR ex20ins in urothelial carcinoma (UC) include CDKN2A/B alterations (55%) and PIK3CA alterations (11%) (6). The cooccurrence of EGFR amplification in advanced EGFR ex20ins non-small-cell lung cancer (NSCLC) is associated with shorter progression-free survival (PFS) when treated with amivantamab (18). Furthermore, activation of the PIK3CA/AKT pathway confers resistance to osimertinib in EGFR-mutated advanced NSCLC (19). Use of pemetrexed-based chemotherapy combined with amivantamab may effectively target urothelial carcinoma clones that are intrinsically resistant to amivantamab. Amivantamab is a bispecific antibody that targets EGFR and MET, exhibits immune cell-directing activities, and causes receptor degradation. The Fc-dependent activity of amivantamab is promoted by macrophage- and monocyte-mediated trogocytosis, which is an essential mechanism for antibody-directed receptor downregulation and the elimination of tumour cells (20). These mechanisms have the potential to enable amivantamab to achieve superior efficacy compared with earlier generations of EGFR tyrosine kinase inhibitors. Amivantamab monotherapy achieved an overall response rate of 40% in patients with EGFR exon 20 insertion NSCLC following prior platinum-based chemotherapy (8). In the PAPILLON trial, amivantamab combined with carboplatin and pemetrexed achieved a progression-free survival of 11.4 months as first-line treatment for patients with advanced NSCLC exhibiting EGFR exon 20 insertions and was granted approval by the Food and Drug Administration (FDA) (7). The safety and tolerability of amivantamab in combination with chemotherapy have been established in patients with EGFR-mutant NSCLC (7, 21). In general, combination anticancer therapy can demonstrate efficacy by exhibiting independent drug action with or without a synergistic mechanism (22). Combining chemotherapy with a bispecific antibody could enhance immune cell activation by modulating the tumour microenvironment and overcoming immune suppression (23). A recent case report describes a young woman diagnosed with metastatic urothelial carcinoma harbouring an uncommon deletion in exon 19 of the EGFR gene (Ala750_Ile759delinsGlyGly) with primary resistance to platinum-based polychemotherapy, achieving a 5-month duration of response to osimertinib (24). Prospective studies in larger cohorts are needed to elucidate the role of EGFR-targeted therapies in urothelial carcinoma. A bispecific antibody-drug conjugate, izalontamab brengitecan, targeting EGFR and HER3, showed encouraging antitumour activity with a favourable safety profile in patients with locally advanced or metastatic urothelial carcinoma in a phase II trial (25).

This case illustrates the crucial role of next-generation sequencing in guiding personalised therapeutic strategies for our patient with heavily pretreated and refractory upper tract urothelial carcinoma following exhaustion of conventional treatment options. Moreover, although immunohistochemical staining of squamous cell carcinoma from lung metastasis raises a possibility of primary lung cancer, the concordant genomic profile with the primary nephroureterectomy tissue supports its origin as a metastasis from UTUC. Furthermore, next-generation sequencing of the lung metastasis biopsy on progression did not reveal any mechanisms of acquired resistance. Pan-cancer whole-genome sequencing showed that metastatic tumours display an increase in the total number of driver gene alterations compared with primary tumours in a cancer-type- and treatment-specific fashion (26). Whilst somatic tumour testing assays, including FGFR3 alterations, MMR status, and HER2 IHC, should be performed at the diagnosis of advanced urothelial carcinoma, the timing and role of comprehensive molecular profiling in metastatic urothelial carcinoma should be individualised (27, 28). Biopsy of the lung metastasis and molecular profiling were performed upon disease progression, which might help to identify resistance driver genes. Since metastatic upper tract urothelial carcinoma with squamous cell differentiation is a unique entity of bladder cancer, it is also reasonable to perform a biopsy of the lung metastasis and comprehensive molecular profiling early on at the diagnosis of metastatic disease, which would enable us to sequence our treatment options more strategically. It is a prime time for us to reconsider our classification of tumours, shifting from an organ- to a molecular-based approach. The commentary authored by Andre et al. in Nature contends that categorising malignancies based on their molecular attributes will accelerate the availability of effective treatments for millions of patients (29).

## Conclusion

This case highlights the potential role of targeting synthetic lethality in urothelial carcinoma with MTAP loss. It provides a novel therapeutic approach using an EGFR-MET bispecific antibody for targeting metastatic urothelial carcinoma with EGFR ex20ins. Although the efficacy of combining amivantamab with pemetrexed-based chemotherapy cannot be determined from this single-patient case report, it provides valuable insight into the biology and treatment of this rare subset of upper tract urothelial carcinoma. Further translational work is crucial to better understand the pathogenic mechanisms of MTAP loss and EGFR ex20ins in urothelial carcinoma. Over the past decade, molecularly targeted agents and immune checkpoint inhibitors have revolutionised the treatment landscape for urothelial carcinoma. Biomarker research for urothelial carcinoma continues to evolve, progressing towards biomarker-driven therapies for this disease (3). Innovative trial designs, such as tumour-agnostic basket trials to refine EGFR ex20ins targeting strategies in multiple cancers, including urothelial carcinoma, are warranted.

## Data Availability

The original contributions presented in the study are included in the article/supplementary material. Further inquiries can be directed to the corresponding author.
